# Calibration of Reflectance Standards*

**DOI:** 10.6028/jres.080A.057

**Published:** 1976-08-01

**Authors:** W. Budde

**Affiliations:** National Research Council of Canada, Ottawa, Canada K1A OR6

**Keywords:** Barium sulfate, diffuse reflectance spectra, magnesium oxide, opal glass, radiant flux, reflectance standards, standards calibration

## Abstract

Measurements of the diffuse spectral reflectance are usually not made as direct measurement of the incident and the reflected radiant flux but rather as measurements relative to a standard of known reflectance value.

For the calibration of such standards, different methods have been described in the literature:

1. Goniophotometric methods, also called Indicatrix methods or point-by-point methods.
2. Methods based on the Kubelka-Munk theory.
3. Integrating sphere methods according to Taylor, Benford, Sharp-Little, van den Akker, Korte.

Various materials such as magnesium oxide, barium sulfate or opal glass are being used as standards. Their suitability as transfer or as working standards will be discussed.

The results of comparative measurements between some of these methods will be given.

## I. Introduction

Measurements of the diffuse spectral reflectance have been used for many years as a tool in the measurement of color [1].^1^ However the extensive use of such measurements in analytical chemistry has been developed only recently [2]. In general the measurement of a reflectance requires the measurement of the flux reflected from a surface and that incident on this surface. Such measurements are difficult and therefore in most practical applications only reflectance measurements relative to a standard of known value are being made. The “calibration” of such standards is then done either by a definition or convention (e.g., to agree [5] that the reflectance of smoked MgO = 1.0), or by suitable measurements.

It is the purpose of this paper to outline some of the most important methods for such calibrations, to discuss briefly the physical properties of various materials which are used as standards, and to present a few intercomparative data. In presenting the various methods only the main features will be given and those characteristics which are important for distinguishing the methods and the quantities which are measured. Giving the full theory for each method and all experimental details is beyond the frame of this paper and it must be left to the reader to study the details in the original references.

There are essentially three groups of methods for absolute reflectance measurements:

1. Goniophotometric measurements of the reflectance indicatrix with numerical integration.
2. Methods based on the Kubelka-Munk theory.
3. Methods using integrating spheres.

Although examples for all three groups will be given the emphasis will be on methods in the last group which has many interesting varieties and is most widely used.

## II. Terminology

The terminology in this paper will generally be in accordance with CIE terminology [3] with the following exceptions and additions:

1. The terms radiance and luminance are being used interchangeably. Luminance is used mainly where photometric measurements are involved, while radiance is used in the context of spectral measurements. Geometrically these terms are identical, however luminance refers to values measured with a detector whose spectral sensitivity distribution agrees with the CIE photometric standard observer function.
2. Standards for diffuse reflectance measurements may be calibrated in three different geometries:
  - diffuse/diffuse (*d/d*)
  - normal/diffuse (0*/d*)
  - diffuse/normal (*d*/0).
  The first term always describes the geometry of illumination whereas the second term describes the geometry of the measurement. Diffuse illumination is achieved by means of an integrating sphere. For the measurement of diffusely reflected radiation either a goniophotometer, a hemispherical mirror or an integrating sphere is used.
  For colorimetric measurements the CIE recommends four geometries: 0*/d; d*/0*;* 0/45 and 45/0. For the purposes of this paper the *d/d* geometry is added whereas the 0/45 and 45/0 geometries are not discussed. The reason for this selection is that many techniques in diffuse reflectance spectroscopy are based on the Kubelka-Munk theory which requires the use of an integrating sphere.
3. The term “reflectance” applies only for the *d/d* and the 0*/d* geometry. For the *d*/0 geometry the term “reflectance factor” *β_d/_*_0_ applies which is defined [3] as: ratio of radiant (luminous) flux reflected in the directions limited by the cone to that of the perfect reflecting diffuser identically irradiated (illuminated). If a sample is highly opaque and very matte so that its reflection indicatrix approximates that of a perfect diffuser, then the reflectance factor *β_d/_*_0_ will have the same value as the reflectance *ρ*_0/_*_d_* and the reflectance *ρ_d/d_.* For simplicity the term reflectance will occasionally also be used here for the *d*/0 geometry.
4. In this paper the word “calibration” is used for two different procedures:
  - Calibration of a physical standard means to establish its reflectance value by a suitable procedure. According to the old CIE convention [5] this procedure consisted of preparing a smoked MgO surface and giving this surface the reflectance value 1.0. Now [4, 6] this procedure consists in an absolute reflectance measurement which establishes the reflectance of a physical standard with respect to the perfect reflecting diffuser.
  - Calibration of an instrument for relative measurements refers to the adjustment of this instrument so that correct reflectance values of arbitrary specimens can be determined. For this adjustment a suitable standard with a known reflectance value is used.

## III. Goniophotometric Measurements

This method is also called the “indicatrix-method” or “point-by-point method.” The sample is irradiated with a narrow beam usually in the direction of or near the normal (angle of incidence *ϵ*_1_ ≈ = 0°) and the reflected radiation is measured at various angles (angle of observation 0°≤ *ϵ*_2_≤90^o^) and various azimuths. The incident flux *ϕ*_0_ is determined by measuring either the intensity of the source [7] or the irradiance at the plane of the sample surface [8]. The reflected flux *ϕ_r_* is determined by numerically integrating the measured goniometric values of the radiance [8] or the intensity [7] of the sample.

This method is usually time consuming and difficult. Since the reflected flux must be measured at many angles and the measurement of the incident flux requires a considerable change in the experimental set-up, great care must be taken that source and detector are stable over extended periods of time. The greatest difficulty arises from the fact that the incident flux and the flux reflected into the narrow cone defined by the photometer differ by about 3 to 4 orders of magnitude. This difficulty is usually overcome by using an auxiliary source which provides an intermediate level of signal so that two ratios of about 100 to 1 are to be measured.

This method was used for the earliest diffuse reflectance measurements, strangely enough for determining the reflectance of black materials such as soot [9–11]. However, after integrating sphere methods were developed the goniophotometric method was practically only used for comparison: e.g., McNicholas [12], Taylor [13], Korte [7] and Egan [14] report such comparison of values obtained by goniophotometry or with the integrating sphere method. In recent years Kartachevskaia [15] and Morren [8] used the goniophotometric method by itself to determine reflectances of various materials.

**Figure 1:**
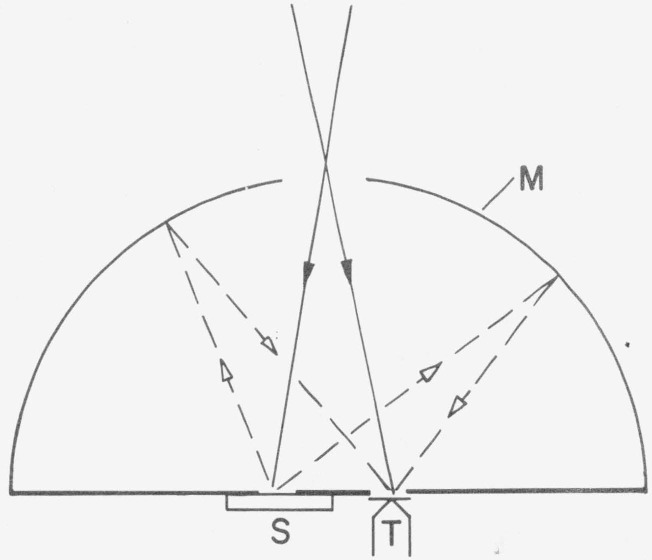
Hemispherical mirror method: M=hemispherical mirror; S = sample; T= detector=thermocouple.

All measurements of the reflected flux at various angles and the integration may be combined into one single step if a hemispherical mirror is used. In figure 1 sample S and detector T are mounted in two closely spaced conjugate points of a hemispherical mirror M. The incident beam is first directed to the detector for the measurement of the incident flux. Then the beam is switched to irradiate the sample. The total flux reflected from the sample is collected by the hemispherical mirror and directed to the detector.

In this technique the reflectance of the hemispherical mirror must be known. It may be determined if a flat sample of the mirror material is mounted at the sample port and a normal measurement is made. The resulting ratio of the two responses is the square of the mirror reflectance.

This technique was used for the earliest reflectance measurements by Rovds, 1910 [10] and Coblentz, 1913 [11] and more recently by Derksen et al in 1957 [16].

The hemispherical mirror technique is not considered to be an integrating sphere technique because it consists in the actual measurement of the ratio of the reflected to the incident flux whereas in integrating sphere techniques the effect of the sample on the radiance of the sphere wall is utilized. This effect is considerably more complex than the simple reflection from the hemispherical mirror.

## IV. Methods Based on the Kubelka-Munk Theory

The Kubelka-Munk theory [17, 18] provides various relations between reflectance values of translucent materials such as powders, sheets of paper, layers of paint over various backgrounds and other physical parameters such as thickness of the layer, “dilution” of a powder with other powders, transmittance, absorption coefficient, etc. These equations suggest certain methods for absolute reflectance measurements which have been tried and reported in the literature.

Stenius [19] measured the reflectance of a sheet of paper over backgrounds of different reflectances relative to a standard and was able to show how the absolute reflectance of the standard used for the relative measurements could be determined from these values (eqs. 12 and 14 of his paper). However it appears that his values are of poor accuracy and he concluded that the Kubelka-Munk equations are not immediately applicable to hand-made sheets of paper. In 1968 A. E. Aneliunas presented a paper at the annual meeting of the Canadian Pulp and Paper Association in which he investigated various aspects of Stenius’ method and proposed a modification which consisted in the measurement of different numbers of sheets of paper thus varying the thickness of the layer by known amounts. From the thicknesses and the associated relative reflectances he determined the scattering coefficient and the absolute reflectance. Although the reflectance values for pressed MgO determined by this method agreed closely with those determined by other methods, indicating the suitability of this method, it was not published in print [63].

A different approach was taken by Butler [20] who was determining absorption coefficients, reflectances and scattering coefficients of various scattering materials from transmittance measurements of layers of various thicknesses. The theory shows that under certain conditions the optical density (=log(1/*T*) where *T* is the measured transmittance) is a linear function of the thickness *X.* The ordinate intercept of this line is related to the reflectance of an infinite layer of the material.

Law and Norris [21] used measurements on small glass spheres as a model particulate system to test the suitability of the method. However in these two papers reflectance values were only a by-product while other parameters such as refractive index, particle size, scattering and absorption coefficients were of major interest. No measurements of the reflectance of commonly used reflectance standards are reported.

The third method was proposed by Lindberg [22] who “diluted” BaSO_4_ powder with Fe_2_O_3_ powder in known concentrations and showed that from these concentrations and the relative reflectances measured for each concentration the absolute reflectance of the standard used for the relative reflectance measurements could be determined. For a pure BaSO_4_ he found values very close to those published elsewhere. However, the method is timeconsuming and it was estimated that the determination of the reflectance “should be good to 0.5 percent” which is a poorer level of accuracy than can be achieved by other methods.

It appears that only the last of these three methods was developed specifically for the calibration of reflectance standards. It was attempted to achieve this with instrumentation which is standard equipment in an analytical laboratory: a spectrophotometer with a reflectance attachment and a weight scale.

Interesting as these methods are their complexity and lack of accuracy and the fact that they are not immediately applicable to all types of reflectance standards seem to have precluded these methods from being adopted by major standardizing laboratories for the calibration of reflectance standards.

## V. Integrating Sphere Methods

In 1912 Nutting [23] published a method for absolute reflectance measurements based on the theory of two parallel infinite planes (which he realized by means of a ring mirror). His method is not of interest and has rarely been used afterwards. His values were discussed and rejected in 1920 when three papers were published on reflectance measurements by means of integrating spheres.

### A. The Taylor Methods

Based on certain considerations developed during the construction of an 88 inch integrating sphere for luminous flux measurements [24], A. H. Taylor in 1920 published a paper [13] in which “five absolute methods of measuring reflection factors are described, at least three of which are apparently new.” One of these methods is for the measurement of specular reflectance and one is a goniophotometric method as described in section III. This latter method was used to verify the values obtained by the sphere methods. The three integrating sphere methods are the “new methods” and are described as follows.

#### First Taylor Method

**Figure 2:**
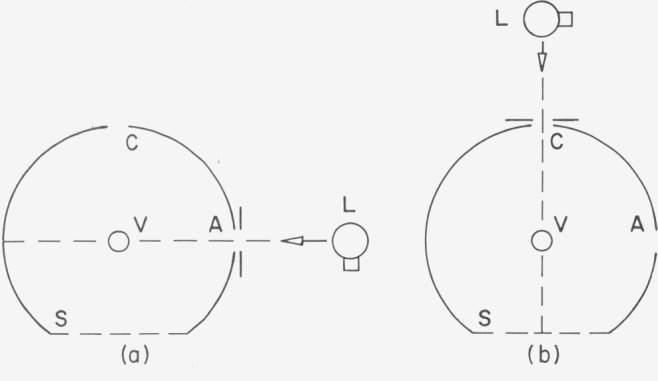
First and second Taylor method: A and C apertures for entering beam; V=window for photometer; S=sample aperture; L= light source.

The integrating sphere, see figure 2a, has two entrance ports A and C and one sample port S which comprises about 10 percent of the total sphere area. Through a viewing port V a photometer views an area on the opposite wall for measurements of the luminance (“brightness” in Taylor’s terminology) of the wall. For this method the light source L remains at port A.

For the determination of the reflectance of the sphere wall two measurements are made

1. The sample port remains open or is covered with a nonreflecting sample. A luminance *B*_0_ is measured.
2. The sample port is covered with a sample having the same paint as the sphere wall. A luminance *B* is measured.

From the measured ratio *k=B/B*_0_ and the known geometrical constants of the sphere (diameter of sphere and sample port) the diffuse-diffuse reflectance *ρ_w_* (“*m*” in Taylor’s eq 9) is calculated. For the determination of the reflectance *ρ*_x_ of an arbitrary sample the sample port is covered with this sample and a luminance *B*_x_ of the sphere wall is measured. From the ratio *k_x_ = B_x_/B*_0_ (where *B*_0_ is the luminance of the wall with S uncovered as before) the geometrical sphere constants, and *ρ_w_*, the reflectance *ρ_x_* is calculated.

This method obviously yields the diffuse/diffuse reflectance of sphere paint and sample. It is necessary to determine the reflectance of the sphere paint, before that of a sample can be determined. In Taylor’s theory the apertures for photometer and entering beam are considered negligibly small. Preston [25] and Middleton and Sanders [26] expanded the theory to make corrections for these apertures and proposed some modifications to the experimental technique. While Taylor and Preston used white light for their experiments, Middleton and Sanders incorporated a monochromator in the apparatus and reported spectral values. Budde [27] used this method with a small sample aperture so that the difference between the flat sample area and the spherical cap over the sample area was negligibly small. Thus the theoretical considerations and the final equations were simplified and a close resemblance to the “fractional sphere method”, see section 5.2, was obtained without the limitation of the fractional sphere method which is applicable to sphere paints only.

#### Second Taylor Method

In this method the reflectance *ρ_w_* of the sphere paint is determined as in the first Taylor method. Then the source L is mounted at the entrance port C, see figure 2b. The sample port is again covered with the flat sample of the sphere paint and a luminance *B'* is measured on the sphere wall. Then the unknown sample is mounted at the sample port and the luminance *B_x_* is measured. From the ratio *R=B_x_/B'*, the geometrical sphere constants, and *ρ_w_*, the reflectance *ρ_x_* is calculated (Taylor’s Eq 16).

In this method the *d/d* reflectance of the sphere paint is determined in the first step but in the second step the 0*/d* reflectance of the flat sphere paint sample is compared with that of the unknown sample. The assumption is that *ρ_d/d_=ρ*_0_*_/d_* for the sphere paint which is only true if the paint is very matte and opaque.

#### Third Taylor Method

Taylor describes this method as follows: “the sample was placed in a sphere and a very narrow beam of light was projected through a small hole in the sphere wall onto the sphere surface at a point unscreened from the observation window, then onto the sample so placed that none of the first reflected light from it could reach the observation window. The ratio of the brightness of the window in the second case to that in the first case is the reflection factor of the test surface.”

**Figure 3:**
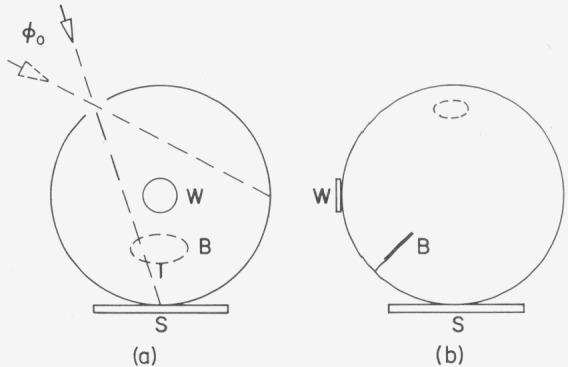
Third Taylor method: ϕ_0_=flux entering the sphere; S=sample; B=baffle; W=observation window. The sphere is shown in two different views to illustrate the location of the baffle.

Taylor gives no drawings for this method but figures 3a and 3b may be used to explain the salient facts. The sphere has a screen B which blocks any direct radiation from the sample S to the observation window W which is probably a ground glass. It also may be the area viewed by a photometer through an aperture in the opposite wall. First the incident flux *ϕ*_0_ irradiates the sphere wall and the observation window receives radiation directly from the irradiated area as well as by multiple reflection from the sphere. Then the source is moved around to irradiate the sample S. The observation window receives only radiation by multiple reflection. The ratio of the two irradiances at the window is the 0*/d* reflectance of the sample.

The essential feature of this method is the introduction of the screen B which blocks the observation window from receiving radiation from the irradiated spot on the sample. It is important to note that in this method it is not necessary to determine first the reflectance of the sphere paint. The measured ratio is not that of sample reflectance to sphere wall reflectance but rather the absolute reflectance of the sample itself.

For this method, Taylor constructed a simple instrument which is described in reference 28. The influence of the apertures and the goniophotometric properties of the sphere paint were investigated by Reule [28a].

It must be mentioned that in the literature the terms “Taylor Method” or “Taylor Sphere” are often used without a clear indication which of the three methods is meant.

### B. The Fractional-Sphere Method

Also in 1920 F. A. Benford published a paper [29] on an integrating sphere method in which the sphere has one or more removable sections. The method is based on the fact that the luminance of the sphere wall depends, among other factors, on the reflectance of the sphere paint and on the actual amount of the wall area if the sphere is incomplete. If the sphere has removable areas so that two fractional spheres with remaining relative wall areas *a*_1_*=A*_1_/4*πr*^2^ and *a*_2_ *= A*_2_/4*πr*^2^ are obtained and if the two luminances or irradiances of the wall measured in the two fractional spheres are *R*_1_ and *R*_2_, the amount of radiation entering the spheres being identical, then it can be shown that the reflectance of the sphere wall is given by the simple relation

$$\rho_{w}=\frac{R_{1}-R_{2}}{a_{1}R_{1}-a_{2}R_{2}}.$$

This method is directly applicable only for materials which can be applied to the inner surface of the sphere and yields the *d/d* reflectance of the material. It has a strong resemblance to the first part of the first Taylor method for the determination of the reflectance of the sphere paint. However in Taylor’s method the removable portion of the sphere is flat and this makes Taylor’s theoretical considerations and the final equation rather complicated. In Benford’s method all removable sections are spherical and the theory and final equation are simple.

In two subsequent papers [30, 31] extensions and applications are described. In 1955 Tellex and Waldron [32] used this method for the determination of the reflectance of electrostatically deposited, smoked MgO.

### C. The Sharp-Little Method

**Figure 4:**
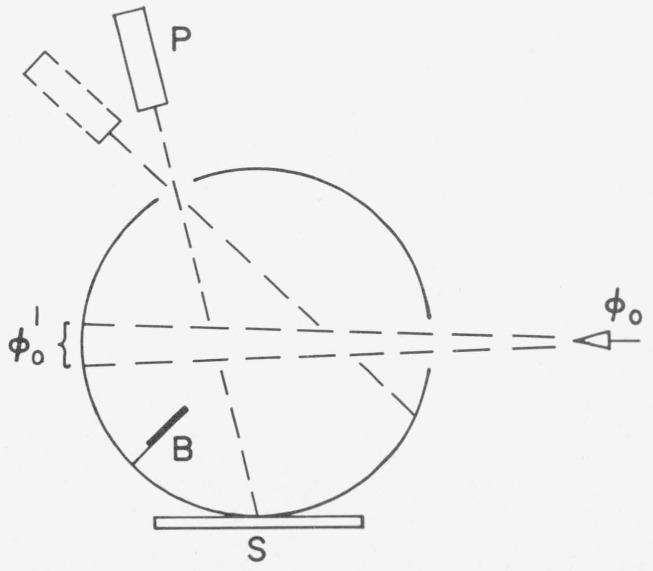
Sharp-Little method: ϕ_0_ flux entering the sphere; ϕ′_0_ = diffuse source within the sphere; B=baffle; S = sample; P = photometer viewing either the sample or the sphere wall.

The third paper on integrating sphere methods published in 1920 was that by Sharp and Little [33]. This method is in fact a geometrical inversion of the third Taylor method and may be explained using figure 4. Flux *ϕ*_0_ enters the sphere and forms an irradiated spot at the wall from which flux *ϕ*′_0_ is reflected. The sample S receives only indirect irradiation from the sphere wall due to multiple reflections because of the screen B while all other areas of the wall receive direct illumination from *ϕ*′_0_ as well as the indirect radiation. The photometer P measures first the luminance *B_s_* of the sample and then the luminance *B_w_* of the unscreened sphere wall; the *d*/0 reflectance *ρ_x_* of the sample is given by *ρ_x_ =B_S_/B_w_.*

Here again the essential part is the baffle B which prevents the direct illumination of the sample from the irradiated area (which may be considered a secondary source within the sphere).

The authors assumed that the apertures for the entering flux and for the photometer were negligibly small. Budde [34] expanded the theory to allow corrections for the apertures and also indicated that the same type of corrections are applicable to the third Taylor method.

**Figure 5:**
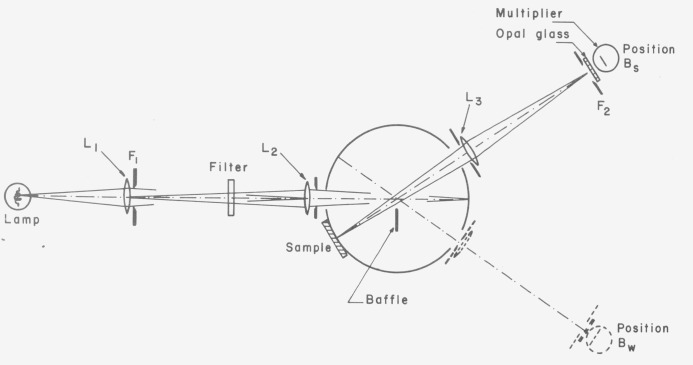
Detailed schematic diagram of an instrument for measurements of ρ_d/0_ (according to Sharp-Little) or ρ_0/d_ (according to the third Taylor method). Explanations in the text.

The schematic diagram of an instrument [35] which may be easily converted from the Sharp-Little geometry to that of the third Taylor method is shown in figure 5. The lamp is imaged by lens L_1_ into lens L_2_ which in turn images the aperture F_1_ onto the sphere wall. The baffle near the center of the sphere blocks any direct radiation from the image (the secondary source within the sphere) to the sample. The “Filter” is one of a set of interference filters in a filter wheel to allow spectral measurements. A photometer system, consisting of lens L_3_, aperture F_2_, an opal glass and a photomultiplier, measures the radiance of the sample *B_s_* and then swings around to measure the radiance *B_w_* of the sphere wall. It can be shown that

$$\rho_{x}=\frac{B_{s}}{B_{w}}\frac{A_{0}}{A_{1}+A_{2}}$$

where *A*_0_=4*πr*^2^ = total sphere area, *A*_1_ = area of sample port and *A*_2_ = area of remaining sphere wall. The factor *A*_0_/(*A*_1_*+A*_2_) represents a correction factor for the entrance aperture and the two observation apertures which are supposed to be black. In this set-up the instrument yields the diffuse/normal reflectance factor according to the Sharp-Little method. It may be easily converted to the inverse geometry by exchanging the source (with lens L_1_) and the detector (with the opal glass). Then it yields the normal/diffuse reflectance according to the third Taylor method.

This instrument has been constructed at the National Research Council of Canada and is being used for the calibration of reflectance standards [35].

### D. The Double-Sphere Method

**Figure 6:**
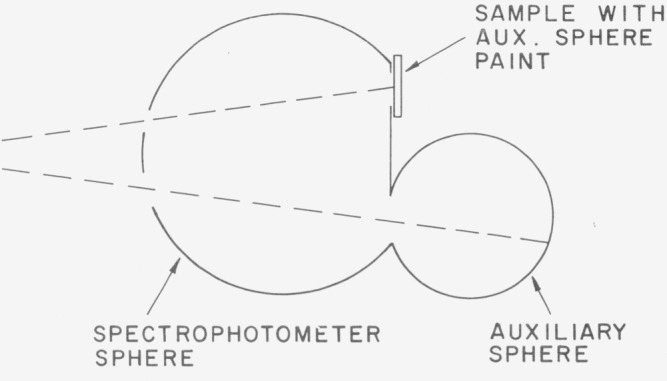
Double sphere method.

In 1966 van den Akker et al. [36] published a paper on absolute spectral reflectance measurements in which an auxiliary sphere is mounted at the sample port of the integrating sphere of a diffuse- reflectance, double-beam spectrophotometer, see figure 6. At the reference port of the spectrophotometer a flat sample is mounted which has the same paint as the auxiliary sphere. If the ratio of the port area of the auxiliary sphere to the total inner area of this sphere is *p* and the ratio of the reflectance of the auxiliary sphere to that of the flat sample (the ratio measured by the spectrophotometer) is *r* then it is shown that the diffuse/diffuse reflectance of the sphere paint, and the flat sample, is given by:

$$\rho_{d/d}=(r-p)/[r(1-p)]$$

An application of this method and some error analysis was published shortly afterwards in a paper by Goebel et al. [37], and this method was also adopted in ASTM and TAPPI Standards.

This method is presently used at the NBS for the calibration of reflectance standards.

### E. The Korte (PTB) Method

**Figure 7:**
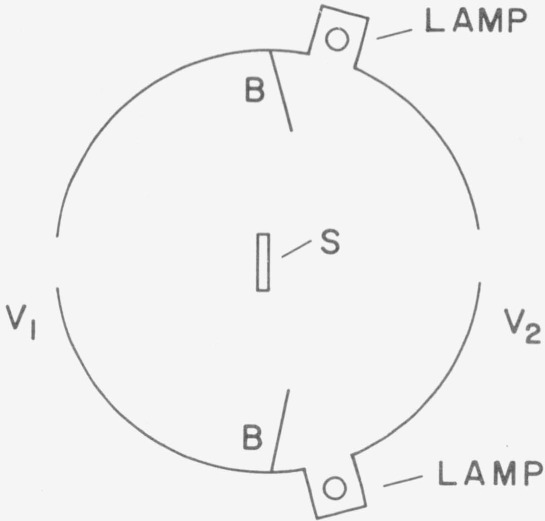
Korte-method: B=baffles; S=sample with front surface facing observation window V_1_; V_1_ and V_2_ = observation windows. For the measurement of the sphere radiance the photometric system is looking past the sample at the sphere wall in the vicinity of window V_1_.

While in all previous methods the samples were mounted at a port of the integrating sphere Korte [7] described a method where the sample is mounted in the center of an integrating sphere. Six lamps are mounted in one hemisphere so that they do not irradiate the sample surface directly. The sample is irradiated only indirectly from the other hemisphere, see figure 7. The ratio of the radiance *L_s_* of the sample observed through window V_1_ to the radiance *L* of the illuminating hemisphere observed through window V_2_ gives the reflectance factor *β_d/_*_0_*=L_s_/L* of the sample which in this case for a matte sample is equal to the reflectance, *ρ*_0_*_/d_.*

The most important property of the instrumental arrangement is the uniformity of the radiance of the irradiating hemisphere. Korte reports that a test was made on this uniformity and that it was better than 0.1 percent. The irradiating hemisphere has an aperture for the photometer. A correction for this aperture, which is a nonirradiating area, must be made.

Erb [38] modified this method by placing just one lamp near the center of the sphere at the back of the sample.

This method was already described in principle by de la Perelle [39]. However Korte refined this method considerably to make it suitable for the calibration of standards at the PTB, Germany. Morren [53] also used this method in addition to his goniophotometric measurements for the extension of the wavelength range to the near infrared.

## VI. Properties of Reflectance Standards

The “primary” standard of reflectance is the perfect reflecting diffuser which reflects all incident radiation (*ρ*=1.0) in a perfectly diffuse or Lambertian manner so that its radiance (luminance) is constant for all angles of viewing or that its radiant (luminous) intensity varies with the cosine of the angle between the normal and the angle of viewing. This theoretical concept will probably never be realized materially but the techniques for the measurement of absolute reflectances provide the calibration of material standards relative to this primary standard.

The selection of a material for calibration as a secondary reflectance standard must take into account not only the physical properties but also the application. The most important physical properties to be considered are

1. *Reflectance value:* in most cases a very high reflectance is desirable so that in relative measurements ratios larger than 1.0 do not occur; however other specific values may be desired, e.g. for the measurement of one type of paper opacity a standard having a reflectance of 0.890 is required.
2. *Goniophotometric indicatrix:* for certain applications a very matte standard is required while in other cases a glossy surface is preferable.
3. *Opacity:* a very high opacity is usually desirable in order to avoid edge losses [59].
4. *Uniformity:* across the surface.
5. *Flatness of the surface:* some instruments are very sensitive to small distances between the sample surface and the plane of the sample port.
6. *Stability:* either short term (days or weeks) or long term (months or years).
7. *Cleanability:* the cleaning of a standard may be necessary, particularly in industrial use, and should not change its reflectance.
8. *Transportability:* a standard may be required to be mechanically stable and rugged enough to be sent by mail.
9. *Absence of:* fluorescence, hygroscopic effects, thermochromic and photochromic effects.
10. *Spectral nonselectivity:* it may be necessary to have a spectrally nonselective standard.

Considering the application or use of reflectance standards two major categories must be distinguished:

- “Transfer Standards” which are used to transfer a calibration (or as it is sometimes expressed, a “scale”) from one instrument to another, for example, from an absolute reflectometer to a commercial instrument for relative measurements.
- “Instrument Standards” which are used as day-to-day “working standards” in a particular instrument or as “master standards” for the periodical checking of a working standard. Ideally instrument standards are calibrated against a transfer standard in the instrument in which they are to be used.

The requirements with respect to most of the above listed physical properties are rather obvious for both categories of standards. However there are some significant differences which must be clearly understood.

Transfer standards which are exchanged between a laboratory with instrumentation for absolute reflectance measurements and a laboratory with an instrument for relative measurements, must be insensitive to differences in the geometric conditions, that is they should maintain their reflectance value.

Obviously a standard for an instrument with a *d*/0 geometry should be calibrated in an absolute reflectometer with the same geometry. However, differences may exist in the diameter of the sphere, the existence or location of a gloss trap, the locations of detector aperture, screens, etc., and the transfer standard should be insensitive to them. This insensitivity to geometric differences requires that the standard be very matte, highly opaque and uniform. These are the most important characteristics of a transfer standard and the accuracy of a calibration will depend mostly on the conformity with these requirements.

Materials which approximate the ideal matte surface together with high opacity and uniformity are: pressed BaSO_4_ plates [40], which according to Billmeyer [41] are Lambertian diffusers for angles of incidence up to 50°, pressed MgO and the Russian opal glass MS–20 [60] with a matte surface. Also carefully prepared BaSO_4_ paint [42, 49] and matte ceramic tiles [35] may be used. Pressed BaSO_4_ tablets are so indifferent against changes in geometry that *β*_*d*/__0_= *ρ*_0__/*d*_=*β*_*d*/8_= ρ_8_*_/d_* [34, 43].

For instrument standards the major requirements are: long term stability and cleanability. The latter property is often achieved by giving the material a glossy surface which is much less liable to collect dirt and easier to clean than a matte surface. Examples of such instrument standards are Vitrolite,^2^ opal glass, enamel tiles, or ceramic tiles. A glossy surface of the Russian opal glass MS–20 has also been used [44].

The differences in the requirements for both categories of standards cannot be emphasized enough. Translucent or glossy standards (such as Vitrolite) should never be used as transfer standards and even a change in the geometry within one instrument such as the introduction of a gloss trap necessitates the recalibration of the instrument standard if it is not matte and opaque.

Various properties of materials, particularly the stability, have been discussed in a paper by Erb [45].

Effects of polarization on the reflectance of materials for standardization purposes have recently been investigated [46–48], and it becomes quite obvious that matte powders do not depolarize incident polarized radiation and that radiation reflected from such surfaces is partially polarized even if the incident radiation is unpolarized. However this is not important for integrating sphere measurements because the geometry is usually symmetrical and polarization effects cancel out.

The stability of BaSO_4_ and other reflectance standard materials or sphere paints was also discussed in a paper by Grum and Luckey [49].

The effect of pressure in the preparation of pressed tablets was investigated by Schatz [50] who concludes that “in general for oxide powders (BaSO_4_, MgO, etc.) the reflectance of the compacts decreased with increasing pressure.”

Some remarks are necessary on the Russian opal glass MS–20 which has been proposed for calibration purposes:

1. A polished surface of this material seems to be adequate as a working standard. A depolished, matte surface may be used as transfer standard only for short periods. Cleaning such a matte surface is very difficult and requires a carefully established cleaning procedure in order to yield repeatable values.
2. The material is fluorescent if irradiated by radiation below 370 nm. This fluorescence appears as a faint orange general emission with occasional limited areas of considerably stronger orange emission. This clearly indicates some non-uniformity in the material which may be seen under grazing angles on a polished surface as a very faint difference in the surface texture. Consequently the material should be inspected for these inhomogeneities under fluorescent light and it should not be used in the UV.

A new material “Halon,” which is a fluorocarbon, has been recently proposed by Grum [61]. However more experiences in practical applications are necessary before its suitability may be established.

## VII. Reflectances of Transfer-Standard Materials

A survey and discussion of reflectance values of smoked MgO and of BaSO_4_ and their properties was given by Budde in 1960 [51]. A very comprehensive literature survey of the properties and reflectances of smoked MgO, pressed MgO, pressed BaSO_4_ and several integrating sphere paints was recently prepared by Erb [52]. A report on an international intercomparison of reflectance measurements organized by CIE Technical Committee 1.2, Photometry and Radiometry was prepared by Kartachevskaia et al [55].

For the purposes of this paper it is interesting to collect in a condensed form values measured according to those methods which are employed by various standardizing laboratories:

1. Goniophotometric methods used at
  - LCE, Belgium [8]
  - PTB, Germany [7]
  - ETL, Japan [56]
  - VNIIM, USSR [15]
  - IEN, Italy [56]
2. Sharp-Little method [34] used at NRC, Canada.
3. Double sphere method [36, 37] used at NBS, USA and by Grum [49].
4. Korte-method [7, 54] used at PTB, Germany and also by Morren [53] at LCE, Belgium.
  Data which allow a comparison between goniophotometric and integrating sphere methods are difficult to find. Only in reference 55 are direct comparisons of measurements made in various laboratories on one material (BaSO_4_) reported. Otherwise Taylor [13] finds satisfactory agreement between the goniophotometric method and his second integrating sphere method whereas Korte [7] states that his goniophotometric values are 0.3 to 0.4 percent higher than his sphere values. However he concludes that the difficulties of the goniophotometric method make it less accurate than his sphere method.

In collecting data from the literature either spectral reflectances or the luminous reflectance are found. For reflectance standards having approximately nonselective spectral reflectance distributions the luminous reflectance is very close or equal to the spectral reflectance at 550 nm. Consequently, if in the following tables only a value in the 550 nm column appears, the original value is a luminous reflectance.

Reflectance data for pressed BaSO_4_ other than the Eastman White Reflectance Standard are given in table 1. In this table all values refer to BaSO_4_ produced by Merck, Product No. 1748, distributed by C. Zeiss [43, 57] except for group 3 which refers to an average for powders of four different suppliers and for group 4b which refers to BaSO_4_ produced by Merck, Product No. 1750 which has a slightly lower reflectance than Merck Product No. 1748 (see ref. 34 where data for both products are given).

Reflectance data for pressed BaSO_4_ sold as Eastman White Reflectance Standard are collected in table 2. No reflectance data determined by the goniophotometric method were found.

These tables show two features:

1. that the largest variation of values occurs at the blue part of the spectrum where the reflectance values start to decrease. This is also the spectral region where the reflectance changes slightly [62] for the first few days after pressing and where UV radiation produces the strongest changes in reflectance [38, 45].
2. The double-sphere method always yields the highest values. There is, of course, a difference between the geometries: the double sphere method yields *d*/*d*-reflectance whereas the two other sphere methods yield *d*/0 reflectances and the goniophotometric methods yield 0*/d* reflectances. Whether the discrepancies are inherent in the methods or are due to effects in the material such as retroreflectance is not known and will be the subject of future investigations.

The measurements given in table 1 and 2 refer to samples which were made by different persons in various institutes and possibly also from different charges. Consequently some variations in the reflectance values must be attributed to sample differences.

A direct comparison without such sample differences between the Korte-method and the Sharp- Little-method as modified by Budde [34] was arranged as follows: four BaSO_4_ samples were pressed at NRC and their reflectances at 457 and 550 nm compared in an Elrepho. It was found that the reflectances of these 4 samples agreed to better than ±.02 percent (about ±.0002 in reflectance). Two of these 4 samples were hand-carried to PTB- Germany for measurements according to the Korte method and two were measured at NRC, Canada, according to the Sharp-Little method. The results are given in table 3. The agreement is rather satisfactory, particularly in view of the fact that different arrangements and theoretical treatments are used in the two methods.

## VIII. Concluding Remarks

1. At several stages in the preceding sections the simplifying assumption was made that the reflectance factor *β_d_*_/0_ is equal to a reflectance *ρ_d_*_/0_ which has the same value as the inverse reflectance *ρ*_0/d_. It must be emphasized that this simplification is not generally permissible and that its applicability must be investigated. Only for very matte and opaque samples is such simplification permissible. Pressed BaSO_4_ seems to be such a substance [34] and also the Russian opal MS–20, if ground matte.
2. With respect to precision and accuracy of reflectance calibrations it is found that a precision of ±0.002 is possible whereas accuracies in the order of ±0.003 are claimed. However the discrepancies between values obtained in the double sphere method on one side or in the Sharp, Little and Korte-method on the other side are larger than these claimed accuracies and therefore only after these discrepancies have been explained can a more general statement on accuracy be made.
3. The question may be asked why absolute measurements have been made mainly in national standardizing institutes and why instrument makers have generally refrained from making instruments which yield absolute values. The answer to this question is that absolute reflectance measurements, in spite of the apparent simplicity of the integrating sphere methods, are complex and accident prone. The design of an absolute reflectometer requires a complete understanding of the parameters wdiich may affect the measurements. And to maintain an absolute reflectometer in perfect working order is often beyond the capabilities of an industrial testing laboratory. The International Organization for Standardization (ISO) has recognized this situation with its efforts to establish international agreement in reflectance measurements by delegating the calibration of transfer standards to a few recognized laboratories and recommending only relative measurements throughout the international paper industry [58]. Preliminary results justify this procedure.

Basically this procedure is used with many measured quantities such as resistance voltage, weight, etc., where standardizing laboratories provide the absolute calibration of standards while in industry only measurements relative to those standards are made. There are many good reasons for applying this procedure also to the complicated field of reflectance measurements.

## Figures and Tables

**Table 1:** Reflectance of pressed BaSO_4_ other than Eastman White Reflectance Standard

	Wavelength (nm)					
		400	500	550	600	700

1. Gonio-methods	0*/d*					
LCE, Morren [53]				0. 983		
ETL [55]				. 986		
2. Sharp-Little method NRC, Budde [34]	*d*/0	0. 976	0. 986	. 985	0. 986	0. 987
3. Double-Sphere method NBS, Goebel [37]	*d/d*	. 983	. 989	. 991	. 990	. 991
4. Korte-method	*d*/0					
(a) PTB, Korte [7]				. 984		
(b) Morren [53]	*d*/0	. 965	. 980	. 982	. 983	. 984
(c) Erb [54]	*d*/0	.975	. 983	. 984	.986	. 987

**Table 2:** Reflectance of pressed Eastman White Reflectance Standard BaSO_4_

			Wavelength (nm)				
			400	500	550	600	700

1.	Sharp-Little method NRC, Budde [34]	*d*/0	0. 986	0. 991	0. 991	0. 991	0. 991
2.	Double Sphere method Grum [49]	*d/d*	. 995	. 998	. 998	. 998	. 997
3.	Korte-method Morren [53]	*d*/0	. 980	. 987	. 987	. 990	.990

**Table 3:** Absolute Spectral Reflectance Factors (d/0) of BaSO_4_—Comparison of NRC and PTB

λ	*β_d_*_/0_(λ)		λ	*β_d_*_/0_(λ)	
	
(nm)	NRC	PTB	(nm)	NRC	PTB
	
370	0. 959	0. 962	480	0. 986	0. 985
380	. 968	. 970	500	. 987	. 986
390	. 974	. 975	550	. 988	. 988
400	. 978	. 979	600	. 988	. 988
420	. 982	. 983	650	. 987	. 988
440	. 985	. 984	700	. 988	. 989
460	. 986	. 985	750	. 988	. 988

## Footnotes

1 Figures in brackets indicate the literature references at the end of this paper.

2 In order to adequately describe materials and experimental procedures, it was occasionally necessary to identify commercial products by manufacturer’s name or trademark. In no instances does such identification imply endorsement by the National Bureau of Standards.
